# Supplementary material for: Edible Flower Species as a Promising Source of Specialized Metabolites
Source: Plants (Basel). 2022 Sep 27;11(19):2529. doi: 10.3390/plants11192529 (PMC9570977; doi:10.3390/plants11192529)
Supplement: Supplementary file 1 [file plants-11-02529-s001.zip › plants-1921838-supplementary.pdf]

plants

Article

Edible Flower Species as a Promising Source of Specialized Metabolites

Mia Dujmović [1], Sanja Radman [2,*], Nevena Opačić [2], Sanja Fabek Uher [2], Vida Mikuličin [1], Sandra Voća [1] and Jana Šic Žlabur [1]

[1] Department of Agricultural Technology, Storage and Transport, University of Zagreb Faculty of Agriculture, Svetošimunska cesta 25, 10000 Zagreb, Croatia
[2] Department of Vegetable Crops, University of Zagreb Faculty of Agriculture, Svetošimunska cesta 25, 10000 Zagreb, Croatia
* Correspondence: sradman@agr.hr

Table S1. Flower species used in study

Picture of flower species	Scientific name	Common name and synonyms	Family
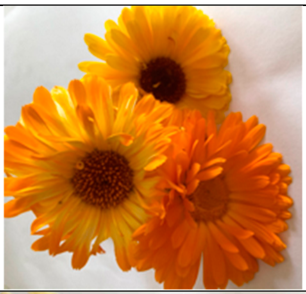	*Calendula officinalis* L.	Common marigold<br>Pot marigold<br>Calendula	Asteraceae
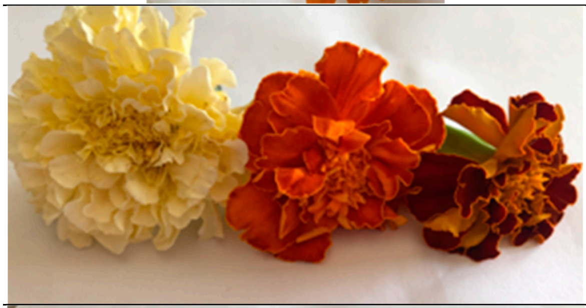	*Tagetes erecta* L.	African marigold<br>Aztec marigold<br>Big marigold	Asteraceae
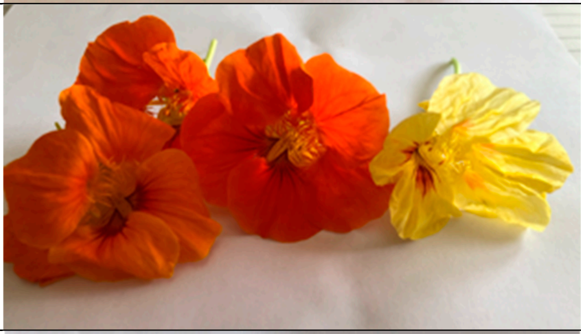	*Tropaeolum majus* L.	Nasturtium<br>Indian cress<br>Monk's cress	Tropaeolaceae
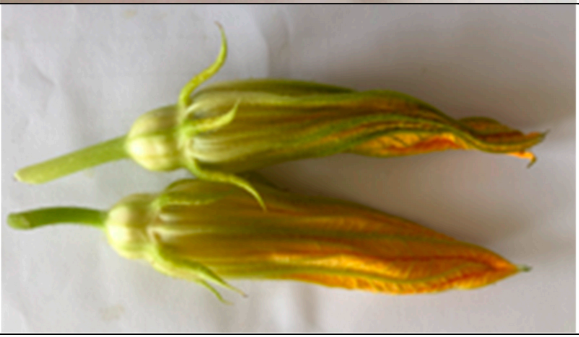	*Cucurbita pepo* L.	Zucchini<br>Pumpkin	Cucurbitaceae
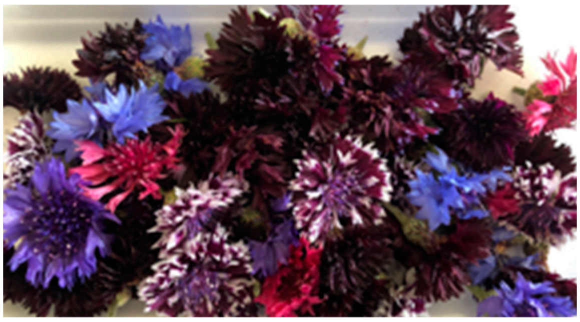	*Centaurea cyanus* L.	Cornflower<br>Bachelor's button	Asteraceae
